# Knowledge Deficit About How Chemotherapy Affects Long-Term Survival in Testicular Tumor Patients

**DOI:** 10.3390/cancers17040565

**Published:** 2025-02-07

**Authors:** Bjoern Thorben Buerk, Charlotte Helke, Emilia Richter, Viktoria Menzel, Angelika Borkowetz, Christian Thomas, Martin Baunacke

**Affiliations:** 1 Department of Urology, University Rostock, 18057 Rostock, Germany; bjoernthorben.buerk@med.uni-rostock.de (B.T.B.); angelika.borkowetz@med.uni-rostock.de (A.B.); 2 Department of Urology, TU Dresden, 01307 Dresden, Germany; charlotte.helke@uniklinikum-dresden.de (C.H.); emilia.richter@uniklinikum-dresden.de (E.R.); viktoria.menzel@uniklinikum-dresden.de (V.M.); christian.thomas@uniklinikum-dresden.de (C.T.)

**Keywords:** testicular cancer, chemotherapy, long-term effects, health service research, survival

## Abstract

Background/Objectives: Patients with germ cell tumors have a good prognosis, which is partly due to platin-based chemotherapy. However, the long-term effects of CTx may play a relevant role in the long-term effects. Up to now, little is known about the knowledge that testicular tumor survivors have about these long-term effects. Methods: A survey of testicular tumor patients treated at the University Hospital Dresden from 2011 up to 2021 was conducted. Patients who had received CTx were asked about their knowledge of the therapy and long-term effects. Results: Of the 279 returns (6 deceased, 46 unavailable, 64% response), 198 patients had received CTx. This was followed up by 63 different urologists. The follow-up period was 5.3 ± 2.9 years. A total of 5% (10/197) did not remember receiving CTx. A total of 55% (102/187) could not name the type of CTx. The most commonly known long-term effects were the risk of second tumors (63%) and andrological problems (59–60%). A total of 18.7% of patients did not know any long-term effects. Half of the patients (54.3%) knew a maximum of 5/14 long-term effects. In a multivariate analysis, a low educational degree (OR 2.2 (1.2–4.3); *p* = 0.02) and one course of carboplatin (OR 3.1 (1.1–9.3); *p* = 0.04) were the independent predictors for a knowledge deficit. Conclusions: Germ cell tumor patients have a low level of awareness of the long-term effects of CTx. This results in a risk of inadequate prevention and follow-up, which, in turn, may have an impact on patients’ long-term survival.

## Simple Summary

This study of testicular tumor survivors treated at the University Hospital Dresden (2011–2021) revealed limited awareness of chemotherapy (CTx) long-term effects. Among the 198 patients surveyed (64% response rate), 55% could not identify their type of chemotherapy, and 18.7% were unaware of any long-term effects. Low education levels and receiving carboplatin treatment were linked to knowledge deficits.

## 1. Introduction

With around 4000 cases per year in Germany, testicular tumors are among the rarer tumors across all cancer entities. However, they are the most commonly malignant tumors in young men in Germany and typically occur between the ages of 25 and 45 [1]. Testicular cancer is a multifactorial disease with known risk factors, such as cryptorchidism or a positive family history of testicular cancer [2]. Good cure rates for metastatic testicular tumors have been achieved with platinum-based combination chemotherapy since the mid-1970s, which were further improved by combining chemotherapies using the BEP regimen (cisplatin, etoposide, and bleomycin) in the mid-1980s [3]. The 5-year survival rate in Germany was most recently reported as 97% [4]. As patients are usually young when combination chemotherapy is administered, and there are high cure rates, late toxicities naturally also play a role in the further course of many patients [5,6]. This is due to the pronounced side effect profile of platinum-based chemotherapies. Late toxicities not only play a role in reducing patients’ quality of life or impairing their daily lives but also decrease life expectancy compared to the general population from around 20 years after chemotherapy [4]. The known side effects of platinum-based chemotherapy include infertility, increased fatigue, cardiovascular and pulmonary toxicity, metabolic side effects, an increased risk of secondary malignancies, as well as nephro-, neuro-, and ototoxicity [7,8]. These side effects are mentioned in the current German testicular carcinoma guideline and should, therefore, be taken into account in follow-ups [9,10]. With regard to the aftercare for long-term effects in testicular tumor patients, two peculiarities can be observed. On the one hand, the patients are closely monitored in urological tumor aftercare. However, the health-related long-term effects primarily concern internal medical conditions, which should be monitored by general practitioners after the tumor aftercare has been completed. On the other hand, the patients are young men who, in their life planning, tend to move house and change doctors more often. Thus, personal responsibility for the early detection of long-term effects is essential, and patients should be aware of these risks. So far, there are no studies regarding the knowledge of germ cell tumor patients on the long-term effects of chemotherapy. Studies on cancer knowledge and awareness of the long-term effects after systemic therapy for childhood cancer show deficits [11,12,13,14]. Our aim was to examine the awareness of testicular tumor patients regarding the long-term effects of chemotherapy.

## 2. Materials and Methods

The participants in this study were patients diagnosed with testicular cancer and treated at the Department of Urology, Medical Faculty Carl Gustav Carus, TU Dresden, between 2011 and 2021. All patients with testicular cancer were included, regardless of specific criteria, including different treatment modalities and oncological stages. We dispatched questionnaires to these participants via postal mail in March 2022. Data collection occurred two months post-questionnaire distribution, with additional follow-up for non-responders for up to two months.

The questionnaire included 26 questions, where we solicited data encompassing sociodemographic parameters (education level, marital status, household income, and type of health insurance) and patterns of information seeking. Furthermore, we asked the patients about their knowledge of therapy and long-term effects. Long-term effects were reported according to the German S3 guideline for testicular tumors (14 long-term effects) [9]. They were asked whether they had been informed about the long-term effects. The answers “No” and “I’m not sure” were categorized as not knowing. The answer “Yes” was counted as knowing. This type of query was based on existing work in the field of pediatric oncological diseases [11,13]. The median of the known number of long-term effects was used to differentiate whether they were categorized as “knowing” or “unknowing”. In this case, only ten long-term effects that significantly impact further aftercare related to prevention were included (excluding problems with ejaculation, testosterone deficiency, infections, and erectile dysfunction). Furthermore, participants were inquired if they recalled having received chemotherapy and whether they knew the name (as free text).

Patients’ records were used to obtain information on the patients’ disease and treatment, such as clinical stage, prognosis group, therapy, and Charlson score. The chemotherapy received was recorded in detail according to the type of chemotherapy and number of series.

Complex chemotherapy was defined as chemotherapy with BEP, cisplatin/etoposide/ifosfamid, or salvage chemotherapy. All patients undergoing complex chemotherapy were treated at our clinic. These patients have all been adequately informed, have received an information sheet about the risks and long-term effects of chemotherapy, and have had a medical consultation about this. Some patients with a history of carboplatin administration were informed by office urologists, and while it can be assumed that they received information, we cannot ascertain the quality of that information.

Data were analyzed using the Chi2-test, the t-test, and a multivariate analysis. Logistic regression models were used for the multivariate estimation of risks and to predict the outcome events.

A *p*-value ≤ 0.05 was considered to indicate significance. All calculations were performed with “IBM SPSS Statistics 28” (Armonk, NY, USA). The study was approved by the local ethics committee (BO-EK-582122021). Ethical approval involved consultation with a data protection officer, ensuring secure data handling.

## 3. Results

### 3.1. Collective

A total of 485 patients with testicular cancer were treated in our clinic between 2011 and 2021. After a mean follow-up of 5.7 ± 3.0 years, 1% (6/485) had died, and 9% (46/485) had moved to an unknown address. The return rate of the questionnaire was 64% (279 out of 433). Of the patients who answered the questionnaires, 198 patients received chemotherapy in the further course. A total of 63 different office urologists treated these patients. The respondents with chemotherapy had a mean age of 44.6 ± 12.2 years. A total of 69% (137/198) had a seminoma, and 31% (61/198) had a non-seminoma. Clinical Stage I accounted for 60% (120/198), Stage II for 25% (49/198), and Stage III for 15% (29/198). Among the respondents who underwent chemotherapy, 35% (70/198) received a single course of carboplatin, while 65% (128/198) received more complex chemotherapy with BEP, PEI, TIP, or salvage chemotherapy (Table 1).

### 3.2. Patient Knowledge

**Figure 1:**
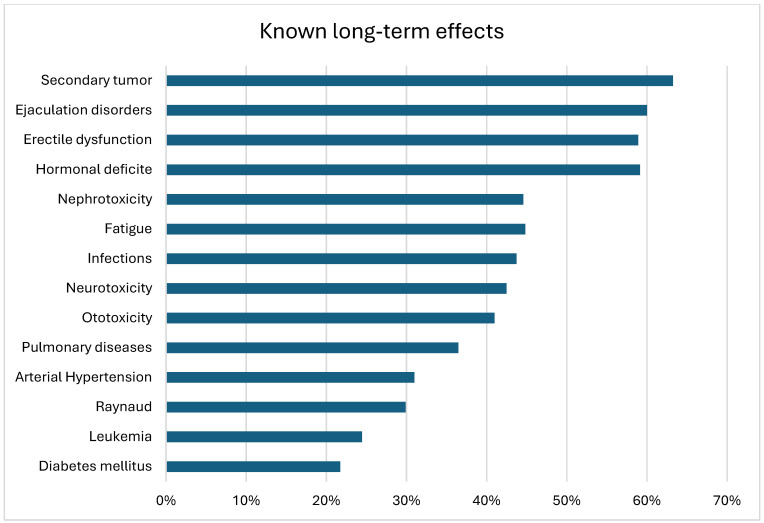
Known long-term effects of chemotherapy (*n* = 186).

**Figure 2:**
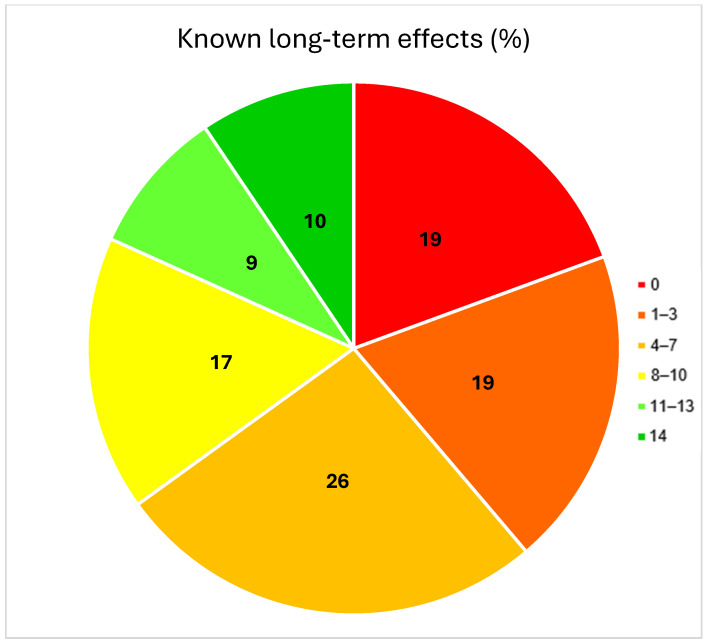
Distribution of the number of the known fourteen long-term effects of chemotherapy (*n* = 186).

A total of 95% (188/198) knew that they received chemotherapy. A total of 45% (85/188) remembered the name of the chemotherapy, 38% (72/188) provided incorrect information about the chemotherapy, and 17% (31/188) could not provide any information on the type of chemotherapy (Table 2). Of all the long-term effects, the best known were secondary tumors (117/185—63%), ejaculation disorders (111/185—60%), erectile dysfunction (109/185—59%), and hormonal deficiency (110/186—59%) (Figure 1). A total of 9.1% stated that they were aware of all of the long-term effects. A total of 18.7% were unaware of any long-term effects (Figure 2). Considering only the relevant long-term effects, 60% (119/198) knew only three or fewer out of ten long-term effects, while 40% (79/198) knew four or more (Table 2).

### 3.3. Risk Factors for Lack of Knowledge About Long-Term Effects

A lack of knowledge was defined by the median of the known relevant long-term effects (three or fewer known long-term effects). Patients with a lack of knowledge had a lower clinical stage (Stage I: 70% vs. 47%, *p* = 0.005), more often a seminoma (76% vs. 59%, *p* = 0.02), more rarely a retroperitoneal lymphadenectomy (13% vs. 27%; *p* = 0.01), more often carboplatin chemotherapy (47% vs. 18%, *p* < 0.001), and fewer chemotherapy cycles (1–2: 47% vs. 18%). Moreover, they also had a lower education level (secondary school or lower: 62% vs. 44%, *p* = 0.01) and were older compared to the patients with more knowledge (46.9 ± 12.5 vs. 41.2 ± 10.9; *p* < 0.001) (Table 3). In the multivariate analysis, the independent predictors for a lack of knowledge on the long-term effects included a low educational level (OR 2.2 (1.2–4.3), *p* = 0.04) and treatment with carboplatin chemotherapy (OR 3.2 (1.1–9.4), *p* = 0.04) (Table 4).

## 4. Discussion

A lack of knowledge about the long-term effects of chemotherapy is a relevant issue in testicular cancer patients. Only 45% (85/188) remembered the name of their chemotherapy. Only 9.1% stated that they were aware of all the long-term effects, and even 18.7% were unaware of any long-term effects. Taking into account only the relevant long-term effects, 60% (119/198) knew only three or fewer out of ten long-term effects. In the multivariate analysis, the independent predictors for a lack of knowledge were a low educational level (OR 2.2 (1.2–4.3), *p* = 0.04) and treatment with carboplatin chemotherapy (OR 3.2 (1.1–9.4), *p* = 0.04).

The current literature on testicular cancer awareness primarily focuses on the topic of self-examination. Numerous studies have explored knowledge about self-examination across various populations and frequently concluded that a deficit exists [15,16]. Even in this cohort of testicular cancer survivors, 24% of those surveyed stated that they did not palpate their remaining testicle at all or only once a year. It is, therefore, not surprising that knowledge about the long-term effects of chemotherapy is very limited. Three studies on this topic have examined the knowledge on the long-term effects in survivors of childhood tumor diseases. A Chinese study with 200 participants revealed low awareness in childhood cancer survivors, showing that only 45% of the participants were aware of more than 25% of the total long-term effects of chemotherapy. This is similar to our findings that 40% of the participants knew more than 30% of the long-term effects. Furthermore, this Chinese study displayed that retaining medical records and better medical information-seeking habits were associated with higher awareness. Interestingly, the level of education had no impact, which may be explained by the fact that 22% of the respondents were not the patients themselves but the parents of underage patients [11]. A further multi-center study from Canada, including 250 survivors of childhood cancers, described that long-term effects with reproductive function were widely known (73%), whereas more problematic long-term effects, like secondary malignancies (45%) and cardiac problems (58%), were less familiar. Independent predictors for knowledge of long-term effects were a younger age and renal cancer survivors. Due to the young age of these patients, no information on their educational level was given [13]. The third study, an American study, including 369 survivors of mainly hematological-oncological tumors, developed an educational concept aimed at increasing the awareness regarding long-term effects. They demonstrated an improvement in awareness as a result of this educational concept. However, the awareness of patients in this study regarding long-term effects before education was similar to the findings in our study, showing good knowledge about fertility problems (63%). In contrast, awareness of more problematic health-related issues, such as renal dysfunction (35%), was underrepresented. Nevertheless, the study aimed to improve awareness by implementing educational lessons [12]. Although based on different cohorts and study approaches, these three studies emphasize the lack of knowledge about long-term effects in cancer survivors.

A lack of knowledge regarding long-term effects can become a significant problem, as long-term effects can not only affect long-term survival but also patients’ quality of life [5,17,18]. It is interesting that long-term effects are best known in relation to testicular function (59–60%). These can affect quality of life but do not have significant health consequences, unlike internal long-term effects. This may be because they are more likely associated by patients with the localization of the tumor. The most commonly known long-term effects, however, are secondary tumors, although it cannot be ruled out that the respondents may have also interpreted this as tumor recurrence.

The question arising from these problems now is how to address the issue. As described in the methodology, all patients with complex chemotherapy were adequately informed about the long-term effects, even though differences in the quality of the information provided by different clinic urologists cannot be ruled out. One of two independent parameters for the lack of knowledge was the administration of carboplatin. In the case of carboplatin, the patient was informed, in part, by office urologists, whose quality of information is unclear. Nevertheless, we believe that the lack of knowledge is more likely due to the less severe nature of the tumor disease and the lower degree of medical intervention (single dose of carboplatin vs. several inpatient stays for PEI or PEB). In view of the very low risk of long-term effects with a single cycle of carboplatin, the relevance of the respondents’ lack of knowledge can also be discussed [19]. The diagnosis of a testicular tumor causes a high level of psychological stress in young patients. Due to the tumor biology, the rapid initiation of therapy is necessary, exposing those affected to a high level of stress [20]. Therefore, we do not expect these patients in this situation to remember the long-term effects of chemotherapy in the context of a medical consultation. Accordingly, the focus of the education on long-term effects should be placed on follow-up care. This is recommended by both the German and European guidelines [9,21]. In our studies, the patients were followed up by 63 different office urologists. In view of the guidelines, it should also be expected that urologists will point this out to patients as part of their follow-up care. Nevertheless, there are aspects that bring the personal responsibility of patients into focus. On the one hand, from a medical point of view, a large proportion of the long-term effects must be monitored by general practitioners or internists. On the other hand, patients are often young and more likely to move and change doctors. Studies show the stress and uncertainty patients experience when transitioning into follow-up care [22,23]. In order to increase patient awareness, they should be specifically educated about the topic of long-term effects through urological aftercare, especially since many long-term effects only appear when tumor aftercare has ended. As a matter of fact, our results demonstrate that a lower educational level is an independent predictor for the lack of knowledge (OR 2.2 (1.2–4.3), *p* = 0.04). Therefore, we propose that this should be considered in patient counselling.

Nevertheless, there are limitations to our study. One relevant issue is the lack of validated questionnaires on the topic of knowledge. We based our questionnaire on previously published studies (Yang, Syed). There is certainly a bias of social desirability when answering the questionnaire regarding the long-term effects since all long-term effects were already listed. However, due to the bias, it can be assumed that the respondents are more likely to tick the wrong box, that they know a long-term effect, than the other way around. Therefore, we assume that without this bias, the knowledge on long-term effects is even poorer. Furthermore, the study is a single-center study. However, this, in turn, allows us to assume that the information provided prior to complex chemotherapy was relatively standardized. For patients receiving carboplatin, this remains unknown. Since the focus is on follow-up, the involvement of 63 office urologists providing aftercare mitigates the limitations of the single-center study. Another limitation is a missing definition of knowledge and a lack of knowledge. We have defined three or fewer out of ten long-term effects as a lack of knowledge. This cut-off was chosen on the basis of a statistical parameter (median). It is debatable whether a higher cut-off could be chosen, especially since it was postulated that a large proportion of the respondents had knowledge deficits. A further limitation of this study is that factors such as perceived self-stigmatization, mental disorders, and other psychological problems, which may promote a knowledge deficit in patients treated with chemotherapy, were not taken into account in this study. However, as this is an unselected patient pool over a longer study period of 10 years, we assume that these factors may play a significant role in individual cases but not in the entire patient collective.

Nevertheless, this is the first study analyzing the knowledge of testicular cancer survivors regarding the long-term effects of chemotherapy. It reveals a crucial supply problem. We have long-term data on the reduced quality of life of testicular tumor patients, allowing us to draw conclusions about the long-term effects of chemotherapy [18]. However, we lack data on whether these long-term effects have occurred despite or as a result of good aftercare and prevention. Although testicular tumor patients in developed countries have excellent 5-year survival rates, long-term survival data indicate a decline in survival rates compared to the general population. It is assumed that the long-term effects of chemotherapy contribute to this trend [24]. It is, therefore, even more crucial that patients are adequately informed about long-term effects and are regularly reminded of them. Given the evidence that appropriate lifestyle changes can help reduce the occurrence of late toxicities, raising awareness about long-term effects has the potential to make a significant difference [25]. It can, therefore, generally be assumed that there is a general lack of knowledge about the possible long-term effects of chemotherapy. On the one hand, we need educational concepts to strengthen patients’ knowledge and awareness. On the other hand, physicians also need to be made more aware of the need to regularly remind chemotherapy patients about follow-up care and the prevention of long-term effects.

## 5. Conclusions

There is a significant lack of awareness regarding the long-term effects of chemotherapy. Particularly among patients who have undergone a less complex chemotherapy and those with a lower level of education. It is a challenge for treating physicians to address this issue and increase patient awareness of the long-term effects in order to improve long-term survival and quality of life.

## Figures and Tables

**Table 1:** Collective of patients with chemotherapy (*n* = 198).

		All (*n* = 198)	Carboplatin (*n* = 70)	Complex Chemotherapy (*n* = 128)	*p*-Value
Age at survey (years)		44.6 ± 12.2 44 (22–81)	48.8 ± 11.6 48 (30–73)	42.3 ± 11.9 40 (22–81)	**<0.001**
Follow-up year (Years)		5.3 ± 2.9 5 (1–12)	5.7 ± 2.7 6 (1–10)	5.0 ± 3.0 5 (1–12)	0.1
Histology	Seminoma	137 (69%)	70 (100%)	67 (52%)	**<0.001**
	Non-Seminoma	61 (31%)	0 (0%)	61 (48%)	
Clinical Stage	I	120 (60%)	69 (100%)	51 (40%)	**<0.001**
	II	49 (25%)	0 (0%)	49 (38%)	
	III	29 (15%)	0 (0%)	29 (22%)	
Prognosis group	Good	60 (78%)	0 (0%)	60 (78%)	n.a.
	Intermediate	9 (12%)	0 (0%)	9 (12%)	
	Poor	8 (10%)	0 (0%)	8 (10%)	
Number of chemotherapy cycles	1–2	103 (52%)	70 (100%)	33 (26%)	**<0.001**
	3+	95 (48%)	0 (0%)	95 (74%)	
Retroperitoneal lymphadenectomy	Yes	36 (18%)	2 (3%)	34 (27%)	**<0.001**
	No	162 (82%)	68 (97%)	94 (73%)	
Educational level (*n* = 192)	Secondary school or no qualification	105 (53%)	36 (54%)	69 (55%)	0.8
	High school	87 (47%)	31 (46%)	56 (45%)	
Marital status (*n* = 197)	Single	66 (34%)	15 (22%)	51 (40%)	**0.01**
	In partnership	131 (66%)	54 (78%)	77 (60%)	
Household income (*n* = 181)	<1500 €	23 (13%)	6 (10%)	17 (14%)	0.2
	1500–4000 €	110 (60%)	35 (55%)	75 (64%)	
	>4000 €	48 (27%)	22 (35%)	26 (22%)	
Health insurance	Statutory	178 (90%)	58 (83%)	120 (94%)	**0.02**
	Private	20 (10%)	12 (17%)	8 (6%)	

**Table 2:** Knowledge of testicular cancer patients with chemotherapy (*n* = 198).

		All (*n* = 198)	Carboplatin (*n* = 70)	Complex Chemotherapy (*n* = 128)	*p*-Value
Chemotherapy according to patient	Yes	188 (95%)	67 (96%)	121 (95%)	0.5
	No	6 (3%)	1 (1%)	5 (4%)	
	I am not aware of	4 (2%)	2 (3%)	2 (1%)	
Knowledge about chemotherapy name (*n* = 188)	Name known	85 (45%)	24 (36%)	61 (50%)	0.2
	Inaccurate/incorrect information	72 (38%)	30 (45%)	42 (35%)	
	Not specified	31 (17%)	13 (19%)	18 (15%)	
Knowledge of the tumor (*n* = 194)	Yes	87 (45%)	32 (48%)	55 (43%)	0.6
	No	107 (55%)	35 (52%)	72 (57%)	
Konwledge of tumor type	No/unclear information	122 (62%)	46 (66%)	76 (59%)	0.3
	testicular tumor	10 (5%)	5 (7%)	5 (4%)	
	Seminoma/Non-Seminoma	66 (33%)	19 (27%)	47 (37%)	
Frequency of palpation of the testicle (*n* = 190)	Daily	20 (10%)	8 (12%)	12 (10%)	1.0
	Weekly	84 (44%)	29 (43%)	55 (45%)	
	Monthly	41 (22%)	15 (22%)	26 (21%)	
	Annually	11 (6%)	4 (6%)	7 (6%)	
	Never	34 (18%)	12 (17%)	22 (18%)	
Knowledge about relevant CTx long-term effects	≤30%	119 (60%)	56 (80%)	63 (49%)	**<0.001**
	>30%	79 (40%)	14 (20%)	65 (51%)	

**Table 3:** Collectively categorized analysis based on knowledge of relevant chemotherapy long-term effects (*n* = 198).

		All (*n* = 198)	Lack of Knowledge (0–30%) (*n* = 119)	Knowledge (40–100%) (*n* = 79)	*p*-Value
Clinical stage	I	120 (60%)	83 (70%)	37 (47%)	**0.005**
	II	49 (25%)	23 (19%)	26 (33%)	
	III	29 (15%)	13 (11%)	16 (20%)	
Prognosis group	Good	60 (78%)	28 (80%)	32 (76%)	0.9
	Intermediate	9 (12%)	4 (11%)	5 (12%)	
	Poor	8 (10%)	3 (9%)	5 (12%)	
Histology	Seminoma	137 (69%)	90 (76%)	47 (59%)	**0.02**
	Non-Seminoma	61 (31%)	29 (24%)	32 (41%)	
Retroperitoneal lymphadenectomy	Yes	36 (18%)	15 (13%)	21 (27%)	**0.01**
	No	162 (82%)	104 (87%)	58 (73%)	
Type of chemotherapy	Carboplatin	70 (35%)	56 (47%)	14 (18%)	**<0.001**
	Complex	128 (65%)	63 (53%)	65 (82%)	
Number of chemotherapy cycles	1–2	103 (52%)	73 (61%)	30 (25%)	**0.001**
	≥3	95 (48%)	46 (39%)	49 (75%)	
Marital status (*n* = 197)	Partnership	131 (66%)	81 (69%)	50 (63%)	0.4
	Single	66 (34%)	37 (31%)	29 (37%)	
Educational level (*n* = 192)	Secondary school or lower	105 (53%)	71 (62%)	34 (44%)	**0.01**
	High school	87 (47%)	43 (38%)	44 (56%)	
Age at interview		44.5 ± 12.2 44 (22–81)	46.9 ± 12.5 48 (22–81)	41.2 ± 10.9 39 (22–71)	**<0.001**
Follow-up year after chemotherapy		5.3 ± 2.9 5 (1–12)	5.5 ± 2.8 6 (1–12)	4.9 ± 3.1 5 (1–12)	0.1
Household income (*n* = 181)	<1500 €	23 (13%)	17 (16%)	6 (8%)	0.3
	1500–4000 €	110 (60%)	64 (59%)	46 (63%)	
	>4000 €	48 (27%)	27 (25%)	21 (29%)	
Health insurance	Statutory	178 (90%)	109 (92%)	69 (87%)	0.3
	Private	20 (10%)	10 (8%)	10 (13%)	
Knowledge of chemotherapy name (*n* = 188)	Yes	85 (45%)	35 (32%)	50 (63%)	**<0.001**
	No	103 (55%)	74 (68%)	29 (37%)	

**Table 4:** Multivariate analysis of predictors for a lack of knowledge regarding chemotherapy long-term effects (*n* = 198).

Parameters	Univariate Analysis		Multivariate Analysis	
	OR	*p*-Value	OR	*p*-Value
Clinical Stage (Stage I)	2.6 (1.5–4.7)	**0.001**	1.7 (0.7–4.1)	0.2
Histology (seminoma)	2.1 (1.1–3.9)	**0.02**	0.9 (0.4–2.0)	0.8
Retroperitoneal lymphadenectomy (no)	2.5 (1.2–5.2)	**0.01**	1.5 (0.6–3.6)	0.4
Type of chemotherapy (Carboplatin)	4.1 (2.1–8.2)	**<0.001**	3.2 (1.1–9.4)	**0.04**
Chemotherapy cycles (1–2 cycles)	2.6 (1.4–4.7)	**0.001**	0.8 (0.3–2.1)	0.6
Educational degree (secondary school or lower)	2.1 (1.2–3.8)	**0.01**	2.2 (1.2–4.3)	**0.02**
Age (>44 years)	2.6 (1.4–4.6)	**0.002**	1.8 (0.9–3.5)	0.1

## Data Availability

The datasets generated during and/or analyzed during the current study are available from the corresponding author on reasonable request.
